# Rapid detection of multidrug-resistant tuberculosis based on allele-specific recombinase polymerase amplification and colorimetric detection

**DOI:** 10.1371/journal.pone.0253235

**Published:** 2021-06-11

**Authors:** Nuntita Singpanomchai, Yukihiro Akeda, Kazunori Tomono, Aki Tamaru, Pitak Santanirand, Panan Ratthawongjirakul

**Affiliations:** 1 Program of Molecular Sciences in Medical Microbiology and Immunology, Department of Transfusion Medicine and Clinical Microbiology, Faculty of Allied Health Sciences, Chulalongkorn University, Bangkok, Thailand; 2 Division of Infection Control and Prevention, Osaka University Hospital, Osaka University, Osaka, Japan; 3 Department of Bacteriology, Osaka Prefectural Institute of Public Health, Osaka, Japan; 4 Microbiology Unit, Faculty of Medicine Ramathibodi Hospital, Mahidol University, Bangkok, Thailand; 5 Research Unit of Innovative Diagnosis of Antimicrobial Resistance, Department of Transfusion Medicine and Clinical Microbiology, Faculty of Allied Health Sciences, Chulalongkorn University, Bangkok, Thailand; Indian Institute of Technology Delhi, INDIA

## Abstract

Multidrug-resistant tuberculosis (MDR-TB) poses a serious threat to TB control. Early diagnosis and proper treatment are essential factors to limit the spread of the disease. The existing molecular tests for MDR-TB usually require specific instruments, steady power supply, and routine maintenance, which might be obstacles for low-resource settings. This study aimed to develop allele-specific isothermal recombinase polymerase amplification (allele-specific RPA) to simultaneously detect the most common mutations in the *rpoB* gene at codons 516, 526, and 531, which are associated with rifampicin resistance, and in the *katG* gene at codon 315, which is related to isoniazid resistance. Allele-specific primers targeting four major mutations, *rpoB*516, *rpoB*526, *rpoB*531, and *katG*315, were constructed and used in individual RPA reactions. The RPA amplicons were endpoints detected by the naked eye immediately after applying SYBR Green I. The optimised RPA assay was evaluated with the *Mycobacterium tuberculosis* wild-type strain H37Rv and 141 clinical *M*. *tuberculosis* isolates. The results revealed that allele-specific RPA combined with SYBR Green I detection (AS-RPA/SYBR) detected these four major mutations with 100% sensitivity and specificity relative to DNA sequencing. The limits of detection for these particular mutations with AS-RPA/SYBR were 5 ng. As a result of the outstanding performance of AS-RPA/SYBR, including its easy setup, speed, lack of a specific instrument requirement, and lack of cross-reaction with other bacteria, this technique may be integrated for the molecular diagnosis of MDR-TB, especially in low-resource settings.

## Introduction

Tuberculosis (TB) remains a significant health problem worldwide. In 2019, the World Health Organization (WHO) estimated that approximately 10 million people developed TB disease and that approximately 1.4 million affected people died [1]. Multidrug-resistant tuberculosis (MDR-TB), defined as *Mycobacterium tuberculosis* that resists at least two of the most potent medications against TB infection, rifampicin and isoniazid, is a challenge for TB control. In 2019, the incidence of multidrug-resistant/rifampicin-resistant tuberculosis (MDR/RR-TB) was estimated to be approximately 465,000 cases, while 206,030 cases were noted with a 57% success rate for treatment outcomes [1]. MDR-TB expands the disease transmission period, leading to increased morbidity, mortality, and treatment failure rates. Over 95% of rifampicin-resistant isolates possess single point mutation clustering in the 81-base-pair hotspot region of the *rpoB* gene known as a rifampicin-resistant determining region (RRDR) that encodes codons 507 to 533. The highest global prevalence of mutations in the RRDR is at codons 531, 526, and 516 [2–4]. In contrast, mutations in several genes can lead to isoniazid resistance. However, the majority (60–80%) of isoniazid resistance in *M*. *tuberculosis* is due to a single point mutation in the *katG* gene at codon 315 (50–93% of *katG* mutations) [2, 5].

The crucial steps to limit TB spread are early detection and appropriate treatment [6, 7]. Molecular diagnostic approaches have been developed for rapid TB diagnosis and drug susceptibility testing. However, several molecular methods mainly rely on amplification-based technologies such as PCR, which are being implemented in developing countries to shorten the turnaround time of conventional phenotypic methods [8, 9]. Xpert MTB/RIF (Cepheid, Sunnyvale, CA, USA) has been introduced to developing countries for the simultaneous detection of TB and rifampicin resistance through cartridge-based fully automated real-time PCR [10]. Although Xpert MTB/RIF is rapid and endorsed by the WHO for initial testing of MDR-TB detection, some limitations, including a reliance on a stable power supply and regular maintenance, impede its potential for application as a point-of-care test (POCT). Furthermore, high-cost laboratory instrumentation and cartridges impact sustainability in the long term in countries with a high TB burden, including Thailand. Other molecular techniques for MDR-TB detection, such as line probe assays, are PCR-based endpoint detection systems [11]. After amplification, the amplicons are detected by test strips, which are lined with a broad set of mutation-specific detection probes that complicate result presentation and hinder their practical use in routine diagnostics. Hence, rapid, easy-setup and low-cost MDR-TB screening systems used as POCTs in low-resource settings are urgently required.

Recombinase polymerase amplification (RPA) has emerged as a novel isothermal amplification technique. With the benefit of two core enzymes (a recombinase and a strand displacing DNA polymerase) and a single-stranded DNA-binding (SSB) protein, RPA does not depend on the thermal denaturation of a template and operates at a low and constant temperature [12]. Thus, thermocycler-independent amplification can be performed using only a low-cost heating box, which is advantageous for application in low-resource settings. RPA has been combined with either real-time- or endpoint-based detection for rapid and visual detection of results, which was recently demonstrated for a potential portable POCT for TB and MDR-TB [13–16].

This study’s main objective was to develop an easy-setup, rapid and affordable screening diagnostic method for MDR-TB. Our method was based on allele-specific RPA and SYBR Green I (SYBR), referred to as AS-RPA/SYBR. RPA was used in an isothermal amplification process with a set of allele-specific primers with intentional mismatches that could discriminate hotspot single point mutations conferring rifampicin and isoniazid resistance. The targets we specified were *rpoB*516, *rpoB*526, and *rpoB*531 for rifampicin resistance and *katG*315 for isoniazid resistance. Our method avoided the requirement of electric-based detection; thus, SYBR Green I was used to interpret amplified RPA products by observing a colour change by the naked eye under visible light. The overall time to detection, i.e., the amplification process to the read-out step, was only 30–40 minutes.

## Materials and methods

### Ethical approval

In this study, *M*. *tuberculosis* genomic DNA was extracted from discarded colonies after a routine diagnostic examination. All of the DNA samples were then fully anonymised with no patient data links to protect patient confidentially before accessed by the researchers and used in the subsequent processes. The study protocol was fully approved by the Research Ethics Review Committee for Research Involving Human Research Subjects, Health Science Group, Chulalongkorn University (Certificate of approval number 036/2018).

### DNA controls

Genomic DNA of the standard *M*. *tuberculosis* reference strain H37Rv (ATCC25618) was used as a wild-type control for the study. Genomic DNA obtained from a clinical *M*. *tuberculosis* strain with known mutations at codons 516, 526, and 531 of the *rpoB* gene and codon 315 of the *katG* gene (confirmed by Sanger DNA sequencing) was used as a mutant control. All genomic DNA was kindly provided by the Microbiology Unit, Faculty of Medicine Ramathibodi Hospital, Mahidol University, Bangkok, Thailand. The control DNA concentrations were measured using a spectrophotometer (NanoDrop 8000, Thermo Scientific, USA). The control DNA was stored at -20°C until use.

### DNA samples

A total of 141 genomic DNA samples extracted from leftover *M*. *tuberculosis* colonies were used in this study. All *M*. *tuberculosis* was isolated from patients (one isolate/patient) from the Microbiology Unit, Faculty of Medicine Ramathibodi Hospital, Mahidol University, Bangkok, Thailand (designated Thailand strain, n = 100) and the Department of Bacteriology, Osaka Prefectural Institute of Public Health, Osaka, Japan (designated Japan strains, n = 41). All *M*. *tuberculosis* samples were initially cultured by the MGIT^TM^ liquid culture system (Becton Dickinson and Company, Franklin Lakes, NJ, USA) and differentiated between *M*. *tuberculosis* complex and nontuberculous mycobacteria by the SD Bioline TB Ag MPT64 assay (Standard Diagnostics, Gyeonggi-do, Republic of Korea). A phenotypic susceptibility test for rifampicin and isoniazid was performed by the MGIT^TM^ liquid culture system.

### DNA sequencing analysis

To analyse mutations associated with rifampicin and isoniazid resistance in all 141 *M*. *tuberculosis* DNA samples, two specific targets, the *rpoB* gene (nucleotide positions 1131–1570) and *katG* gene (nucleotide positions 631–1260), were amplified by conventional PCR. Each reaction contained 1X PCR Buffer, 0.2 μM each forward and reverse primer (Table 1), 200 μM dNTPs, 1.5 mM MgCl_2_ and 1.5 U of *Taq* DNA Polymerase (New England Biolabs, Ipswich, MA, USA). Amplification of both targets was performed with the following conditions: initial denaturation at 95°C for 5 minutes, 35 cycles of amplification (95°C for 1 minute, 55°C for *rpoB* or 63°C for *katG* for 1 minute, and 72°C for 1 minute), and a final extension at 72°C for 7 minutes. *M*. *tuberculosis* H37Rv genomic DNA and sterile distilled water were used as positive and negative controls, respectively. The PCR products of both genes were analysed by 1.5% agarose gel electrophoresis with UltraPower DNA/RNA Safedye (Gellex, Tokyo, Japan) and visualised under an ultraviolet (UV) transilluminator. All the PCR products were sent to Bioneer Sequencing Service Co., Ltd., Republic of Korea, to perform sequence analysis by the conventional Sanger method using an ABI 3730XL DNA Analyser (Applied Biosystems, Foster City, CA, USA). Nucleotide sequences of the *rpoB* and *katG* genes from each of the 141 samples were aligned with the corresponding reference sequence of *M*. *tuberculosis* H37Rv (GenBank accession No. NC_000962) using BioEdit software version 7.2.6.

**Table 1:** Primer sequences for PCR and DNA sequencing.

Genetic regions	Primers	Sequences (5’→3’)	Product sizes (bp)	References
*rpoB*	*rpoB* F	GCTGATCCAAAACCAGATCC	440	This study
	*rpoB* R	ACACGATCTCGTCGCTAACC		
*katG*	*katG* F	AGCGGTAAGCGGGATCTGGAGAA	630	[17]
	*katG* R	CATGTCTCGGTGGATCAGCTTGTA		

### RPA primer design and screening

An RPA assay was developed targeting the most common rifampicin- and isoniazid-associated mutations, which are located at codons 516, 526, and 531 of *rpoB* and codon 315 of *katG*, respectively. To identify TB, the IS1081-F and IS1081-R primers were used to amplify an insertion sequence, IS1081, that is highly specific for *M*. *tuberculosis*. For the *rpoB* gene, *rpoB*-F and *rpoB*-R were designed to amplify the *rpoB* gene as a control. A set of *rpoB* allele-specific primers was designed for specific point mutation detection at codons 516, 526, and 531, which confer rifampicin resistance. These primers included *rpoB*516-F and *rpoB*526-F (used in combination with *rpoB*-R) and *rpoB*531-F (used in combination with *rpoB*-R2). For the *katG* gene, *katG*-F and *katG*-R were designed to amplify the *katG* gene as a control. The *KatG*315-F allele-specific primer (used in combination with *katG*-R) was used for specific point mutation detection at codon 315, which was associated with isoniazid resistance.

RPA primers for the *rpoB* and *katG* gene sets were designed from the reference DNA sequence of *M*. *tuberculosis* H37Rv (GenBank accession No. NC_000962) using the Primer3 program (version 0.4.0; http://bioinfo.ut.ee/primer3-0.4.0/) with a modification for allele-specific primers according to the Yaku-Bonczyk method [18]. The 3’ terminus of each forward allele-specific primer was specific to the wild-type alleles responsible for conferring drug resistance. If the targeted location was wild-type, the allele-specific fragment was amplified. In contrast, if the targeted location contained the mutation, amplification of the allele-specific fragment would be prevented. An additional single-base mismatch at the third base from the 3’ terminus of each forward allele-specific primer was added to improve the discriminatory power between wild-type and mutant (S1 Appendix). These RPA primer candidates contained 29–33 bases and had GC contents ranging from 56% to 62%, as recommended by the RPA manufacturer (TwistDx Limited, Maidenhead, UK). The OligoAnalyser 3.1 program (https://eu.idtdna.com/calc/analyser) and BLAST software (https://blast.ncbi.nlm.nih.gov/Blast.cgi) were used to assess the possibility of secondary structure formation and the specificity of the primers, respectively.

Two to four forward and reverse primers for each target were designed in this study. Each forward and reverse primer candidate was paired and preliminarily screened for its ability to amplify specific targets with a TwistAmp® Basic Kit (TwistDx Limited, Maidenhead, UK) as recommended by the manufacturer. Once the most suitable primers were identified, they were finally used in our developed RPA assay. Table 2 summarises all the final selected RPA primers used in this study.

**Table 2:** RPA primer sequences and optimum conditions used in this study.

Genetic region	Primer	Sequence (5’→3’)	Product size (bp)	Reference	Final DNA concentration (ng)	Final primer concentration (μmol/L)
IS1081	IS1081 F	CCTCTTCTCATCTTATCGACGCCGAGCAGC	173	[16]	0.1	0.48
	IS1081 R	CTGATTGGACCGCTCATCGCTGCGTTCGC				
*rpoB*	*rpoB* F	TCGGCGAGCTGATCCAAAACCAGATCCGGGTCG	363	This study	0.1	0.4
	*rpoB*516 F	TCGGCACCAGCCAGCTGAGCCAATTCATCGA	213		0.1	0.5
	*rpoB*526 F	CCAGAACAACCCGCTGTCGGGGTTGACTCA	182		0.1	0.5
	*rpoB* R	CCGACAGCGAGCCGATCAGACCGATGTTGGGC				
	*rpoB*531 F	TCGGGGTTGACCCACAAGCGCCGACTCTC	250		0.2	0.5
	*rpoB* R2	ACACGATCTCGTCGCTAACCACGCCGTCG				
*katG*	*katG* F	CTGATCGTCGGCGGTCACACTTTCGGTAAGACCC	276	This study	0.1	0.2
	*katG*315 F	CCGGAACCGGTAAGGACGCGATCACCGG	152		0.1	0.5
	*katG* R	CTTGGCGGTGTATTGCCAAGCGCCAGCAGGGC				

### AS-RPA/SYBR optimisation

To determine the most appropriate conditions for RPA amplification and SYBR Green I detection, the optimised conditions were validated using genomic DNA of *M*. *tuberculosis* H37Rv (as a wild-type control) and *M*. *tuberculosis* strains with known mutations within the *rpoB* and *katG* genes (as mutant controls). During RPA assay development, the amount of DNA template (0.00005–0.2 ng), primer concentration (0.2–0.6 μM pM), MgOAc concentration (14–20 mM), incubation temperature (37–42°C) and incubation period (15–40 minutes) were optimised until the most appropriate conditions were obtained. SYBR Green I (250-1000X in a final volume of 25 μL of RPA products) was also validated to obtain the appropriate concentration. In every optimisation, the RPA amplicons were examined by SYBR Green I and compared with agarose gel electrophoresis. When observed with SYBR Green I, the condition giving clear readout results without nonspecific bands upon agarose gel electrophoresis was chosen.

### AS-RPA/SYBR assay to detect *rpoB* and *katG* mutations

All 141 *M*. *tuberculosis* DNA samples were amplified by the RPA assay using a TwistAmp® basic kit as recommended by the manufacturer. Genomic DNA of *M*. *tuberculosis* H37Rv and *M*. *tuberculosis* strains with known mutations within the *rpoB* and *katG* genes were used as wild-type and mutant controls, respectively. Amplification of the targets IS1081, *rpoB*, *rpoB*516, *rpoB*526, *rpoB*531, *katG*, and *katG*315 was performed separately. Each RPA reaction was composed of 29.5 μL of RPA rehydration buffer, 14 mM MgOAc, forward and reverse primers (Table 2), DNA template, and sterile distilled water up to 50 μL. The RPA reactions were incubated at 37°C for 15 minutes. At the end of the amplification, RPA amplicons were equally divided into two tubes, 25 μL each, for different endpoint detection methods.

The naked-eye detection of RPA amplicons was performed by immediately observing colorimetric changes in natural light after adding 1 μL of 375x SYBR Green I (TaKaRa Bio, Tokyo, Japan) to 25 μL of RPA amplicons. In the presence of RPA amplicons, the colour of the solution changed from the original orange to bright green, indicating that *M*. *tuberculosis* DNA was detected or that no single point mutation occurred at the specific codons (indicating that the isolate was probably a susceptible wild-type strain). On the other hand, the solution remained orange in the absence of the RPA amplicon, implying that *M*. *tuberculosis* DNA was not detected or that a single point mutation may have occurred at specific codons (indicating that the isolate was probably a resistant mutant strain). The interpretation of RPA amplification coupled with SYBR Green I detection was conducted independently by two different investigators who were blind to the results of the other test to prevent bias by the two different investigators. For a precise comparison of the results, another 25 μL of the RPA amplicons was purified by FavorPrep Gel/PCR Purification (Favorgen Biotech Corp., Ping-Tung, Taiwan). The purified RPA amplicons were electroporated by 1.5% agarose gel electrophoresis with UltraPower DNA/RNA Safedye (Gellex, Tokyo, Japan) and visualised under UV light. All experiments were performed in triplicate.

### Limit of Detection (LOD)

The lowest DNA concentration that was detected by AS-RPA/SYBR was assessed. Genomic DNA of *M*. *tuberculosis* H37Rv (ATCC25618) and clinical *M*. *tuberculosis* strains with known mutations within the *rpoB* and *katG* genes (obtained from the Microbiology Unit, Faculty of Medicine Ramathibodi Hospital, Mahidol University, Bangkok, Thailand, with no patient data links) was serially diluted tenfold from 5 to 0.00005 ng. Each DNA dilution was used as a template for each primer set for RPA amplification. Sterile distilled water was used as a negative control. After amplification, each RPA product was detected by both SYBR Green I and 1.5% agarose gel electrophoresis.

### Specificity testing

To analyse the specificities of AS-RPA/SYBR, genomic DNA extracted from pathogens that commonly cause respiratory tract infection and other common *Mycobacterium* spp. was used as a template. A total of 5 ng of DNA from the individual pathogen was used for each primer set for RPA amplification. These pathogens included clinical *Acinetobacter baumannii*, *Haemophilus influenzae*, *Klebsiella pneumoniae*, *Moraxella catarrhalis*, *Pseudomonas aeruginosa*, *Streptococcus pneumoniae*, *Streptococcus pyogenes*, *Mycobacterium avium*, and *Mycobacterium intracellulare* strains. All of the above pathogens were kindly provided with no patient data links by the Microbiology Unit, Faculty of Medicine Ramathibodi Hospital, Mahidol University, Bangkok, Thailand, and confirmed for species using biochemical testing and MALDI-TOF MS, except for *M*. *avium* and *M*. *intracellulare* that confirmed by a 16S rRNA sequencing. *M*. *tuberculosis* H37Rv (ATCC25618) genomic DNA and sterile distilled water were used as positive and negative controls, respectively.

## Results

### Distribution of drug-resistant phenotypes among *M*. *tuberculosis* samples

Results of the phenotypic susceptibility tests of 141 *M*. *tuberculosis* isolates were provided by the Microbiology Unit, Faculty of Medicine Ramathibodi Hospital, Bangkok, Thailand, and the Department of Bacteriology, Osaka Prefectural Institute of Public Health, Osaka, Japan. These isolates included 73 strains that were rifampicin and isoniazid susceptible, 4 strains that were rifampicin monoresistant, 14 strains that were isoniazid monoresistant, 31 strains that were MDR-TB, and 19 strains that were extremely drug-resistant tuberculosis (XDR-TB) (Table 3).

**Table 3:** Phenotypic drug susceptibility testing.

Strains	Susceptible	Rifampicin monoresistant	Isoniazid monoresistant	MDR-TB	XDR-TB	Total
Thai	73	4	14	9	0	100
Japanese	0	0	0	22	19	41
Total	73	4	14	31	19	141

### Sequencing analysis

Mutations conferring rifampicin and isoniazid resistance were explored by conventional Sanger DNA sequencing of the *rpoB* and *katG* genes, respectively. Almost all rifampicin- and isoniazid-susceptible strains did not possess mutations either in the *rpoB* or *katG* genes, except for one strain that was rifampicin susceptible and had a mutation at *rpoB* codon 533 and one strain that was isoniazid susceptible and had a mutation at *katG* codon 315. Forty-six of 54 (83.19%) strains resistant to rifampicin (rifampicin monoresistant, MDR-TB or XDR-TB) possessed nucleotide mutations within the *rpoB* gene at the top three highest prevalence codons, codons 531 (28 strains, 51.85%), 516 (10 strains, 18.52%), and 526 (8 strains, 14.81%) (Table 4). Additional uncommon mutations within the *rpoB* gene included those at codon 511 (1 strain, 1.85%), codon 513 (2 strains, 3.70%), codon 522 (1 strain, 1.85%), and codon 533 (3 strains, 5.56%). Two rifampicin-resistant strains had no mutations observed within the 81-base-pair hotspot region of the *rpoB* gene. Mutations of *katG* conferring isoniazid resistance (isoniazid monoresistant, MDR-TB or XDR-TB) were found in 38 (59.38%) strains (Table 5), all of which possessed amino acid substitutions at codon 315 (38 strains, 100%). Interestingly, 26 (40.62%) isoniazid-resistant strains did not have a mutation within the *katG* gene.

**Table 4:** Frequency of amino acid substitutions within 81-base-pair hotspot region of the *rpoB* gene of 141 *M*. *tuberculosis*.

*rpoB* gene			
codons	Amino acid substitutions	Frequencies	
		Susceptible to rifampicin	Resistance to rifampicin
No mutation		86	2
511	Leucine → Proline	0	1
513	Glutamine → Proline	0	2
516	Aspartic acid → Valine	0	7
	Aspartic acid → Tyrosine	0	1
	Aspartic acid → Glycine	0	1
522	Serine → Glutamine	0	1
526	Histidine → Tyrosine	0	2
	Histidine → Aspartic acid	0	3
	Histidine → Arginine	0	2
	Histidine → Serine	0	1
531	Serine → Leucine	0	28
533	Leucine → Proline	1	2
516 and	Aspartic acid → Valine	0	1
533	Leucine → Proline		
	Total (n = 141)	87	54

**Table 5:** Frequency of amino acid substitutions within the *katG* gene of 141 *M*. *tuberculosis*.

*katG* gene			
codons	Amino acid substitutions	Frequencies	
		Susceptible to isoniazid	Resistance to isoniazid
No mutation		76	26
315	Serine → Threonine	1	37
	Serine → Asparagine	0	1
	Total (n = 141)	77	64

### Establishment of AS-RPA/SYBR assay

**Fig 1:**
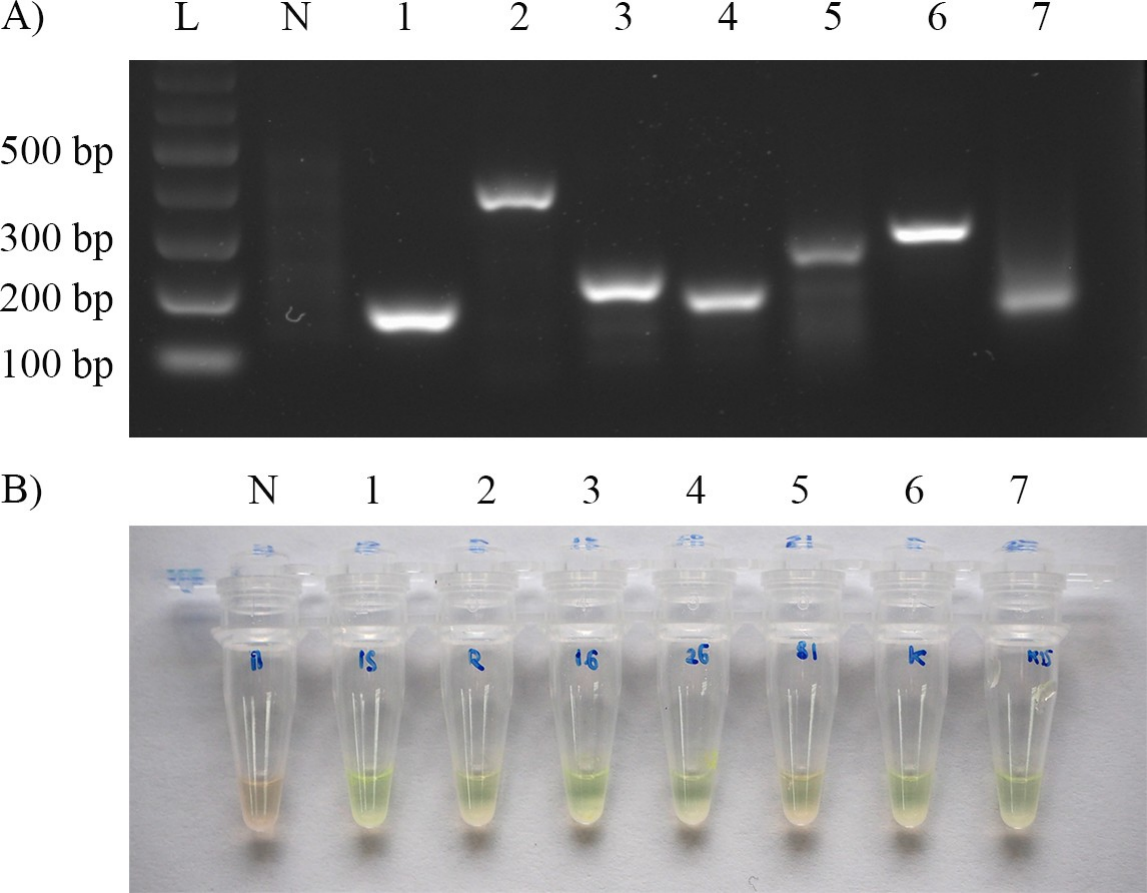
Results of the AS-RPA/SYBR assay validated with the *M*. *tuberculosis* wild-type strain. (A) RPA amplicons were detected via agarose gel electrophoresis. The product sizes from each pair of primers were 173 bp, 363 bp, 213 bp, 182 bp, 250 bp, 276 bp and 152 bp for the IS1081, *rpoB*, *rpoB*516, *rpoB*526, *rpoB*531, *katG* and *katG*315 primers, respectively. Lane L: 100-bp DNA ladder, Lane N: no template control; Lane 1: IS1081 primer; Lane 2: *rpoB* primer; Lane 3: *rpoB*516 primer; Lane 4: *rpoB*526 primer; Lane 5: *rpoB*531 primer; Lane 6: *katG* primer; Lane 7: *katG*315 primer. (B) The naked-eye endpoint detection method was performed by adding SYBR Green I directly to the reaction tubes. Tube N is a no-template control; tubes 1 to 7 contained the IS1081, *rpoB*, *rpoB*516, *rpoB*526, *rpoB*531, *katG* and *katG*315 primers, respectively, showing a green colour, which implied a positive amplification result and no mutation at those allele-specific sites.

**Fig 2:**
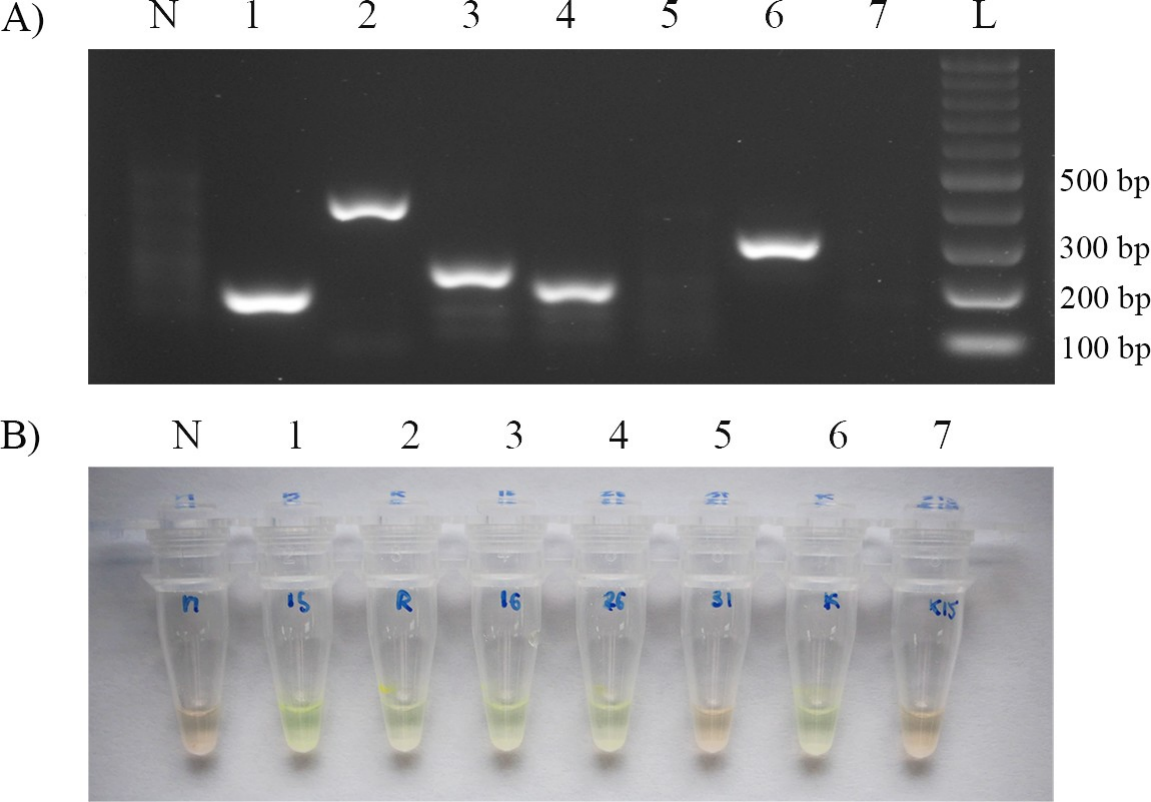
Results of the AS-RPA/SYBR assay when validated with *M*. *tuberculosis* strains with known mutations within the *rpoB* and *katG* genes. This figure represents the results after testing with *M*. *tuberculosis* DNA containing the *rpoB*531 and *katG*315 mutations. (A) RPA amplicons from different primers were detected via agarose gel electrophoresis. The product sizes from each pair of primers were 173 bp, 363 bp, 213 bp, 182 bp and 276 bp for the IS1081, *rpoB*, *rpoB*516, *rpoB*526 and *katG* primers, respectively. Reactions with the *rpoB*531 and *katG*315 primers showed no visible target band. Lane N: no-template control; Lane 1: IS1081 primer; Lane 2: *rpoB* primer; Lane 3: *rpoB*516 primer; Lane 4: *rpoB*526 primer; Lane 5: *rpoB*531 primer; Lane 6: *katG* primer; Lane 7: *katG*315 primer; Lane L: 100-bp DNA ladder. (B) The naked-eye endpoint detection method was performed by adding SYBR Green I directly to the reaction tubes. Tube N was a no-template control; tubes 5 and 7 were tested for *rpoB*531 and *katG*315, respectively, showing an orange colour, which implied a negative amplification result. For allele-specific primers, these results also indicated that the DNA template carried mutations at a particular site. Tubes 1–4 and 6 contained the IS1081, *rpoB*, *rpoB*516, *rpoB*526 and *katG* primers, respectively, showing a green colour, which implied a positive amplification result and no mutation at those allele-specific sites.

After various optimisations, the most appropriate condition for the AS-RPA/SYBR assay was established. The assay included eight individual RPA reaction tubes. The optimised assay showed that AS-RPA/SYBR was able to correctly amplify specific targets and efficiently differentiate the wild-type and mutant templates. When validated with genomic DNA of *M*. *tuberculosis* H37Rv (Fig 1), SYBR Green I remained orange in a negative control tube due to no template DNA for amplification. In contrast, SYBR Green I turned bright green in the remaining tubes, indicating that DNA products were amplified by RPA with specific primer sets. These test results indicate the detection of rifampicin- and isoniazid-susceptible TB. However, when validated with *M*. *tuberculosis* strains with known mutations within the *rpoB* and *katG* genes (Fig 2), SYBR Green I remained orange in a negative control tube and *rpoB*531 and *katG*315 reaction tubes due to the targeted location containing the mutation that inhibited amplification. SYBR Green I turned bright green in the rest of the tubes. These test results indicate the detection of rifampicin- and isoniazid-resistant TB (or MDR-TB). The presence of unaccounted faint smear bands on an agarose gel in Fig 2A (lanes N, 5, and 7) did not lead to a false positive or background on SYBR Green I detection and did not interfere with the assay.

### Detection of *rpoB* and *katG* mutations using the AS-RPA/SYBR assay

The AS-RPA/SYBR assay was evaluated using 141 *M*. *tuberculosis* DNA samples. The AS-RPA/SYBR assay results showed that 140 *M*. *tuberculosis* strains were positive with the IS1081 primers, indicating TB detection, while only one strain was undetectable. However, the *rpoB* and *katG* primer sets enabled the detection of all 141 *M*. *tuberculosis* strains. For the allele-specific primer sets, 10, 8, 28, and 39 strains were negative with the *rpoB*516, *rpoB*526, *rpoB*531, and *katG*315 primer sets, indicating that a single point mutation occurred at the specific codons (rifampicin and/or isoniazid resistance was detected).

Finally, the AS-RPA/SYBR results were validated by DNA sequencing and a phenotypic drug susceptibility test. When compared with the sequencing results (Table 6), both the sensitivity and specificity of AS-RPA/SYBR assay were 100% for detecting codon 516 (95% CI: 69.15–100% and 95% CI: 97.22–100%), codon 526 (95% CI: 63.06–100% and 95% CI: 97.26–100%) or codon 531 (95% CI: 87.66–100% and 95% CI: 96.79–100%) mutations of *rpoB* or codon 315 (95% CI: 90.97–100% and 95% CI: 96.45–100%) mutation of *katG*.

**Table 6:** Validation of AS-RPA/SYBR by a DNA sequencing analysis (N = 141).

AS-RPA/SYBR assay	DNA sequencing		Sensitivity	Specificity
	Mutation detected	Mutation not detected		
*rpoB*516				
Mutation detected	10	0	100% (95%CI: 69.15%-100%)	100% (95%CI: 97.22%-100%)
Mutation not detected	0	131		
*rpoB*526				
Mutation detected	8	0	100% (95%CI: 63.06%-100%)	100%(95%CI: 97.26%-100%)
Mutation not detected	0	133		
*rpoB*531				
Mutation detected	28	0	100% (95%CI: 87.66%-100%)	100% (95%CI: 96.79%-100%)
Mutation not detected	0	113		
*katG*315				
Mutation detected	39	0	100% (95%CI: 90.97%-100%)	100% (95%CI: 96.45%-100%)
Mutation not detected	0	102		

However, when compared with the phenotypic drug susceptibility test results (Table 7), the sensitivity and specificity of the AS-RPA/SYBR assay were 85.19% (95% CI: 72.88–93.38%) and 100% (95% CI: 95.85–100%), respectively, for rifampicin resistance detection. However, the sensitivity and specificity of the AS-RPA/SYBR assay were 59.38% (95% CI: 46.37–71.49%) and 98.70% (95% CI: 92.98–99.97%), respectively, for isoniazid resistance detection.

**Table 7:** Validation of AS-RPA/SYBR by a phenotypic drug susceptibility test (N = 141).

AS-RPA/SYBR assay	Rifampicin or Isoniazid susceptibility tests		Sensitivity	Specificity
	Resistant	Susceptible		
Either *rpoB*516 or *rpoB*526 or *rpoB*531				
Mutation detected	46	0	85.19% (95%CI: 72.88%-93.38%)	100% (95%CI: 95.85%-100%)
Mutation not detected	8	87		
*katG*315				
Mutation detected	38	1	59.38% (95%CI: 46.37%-71.49%)	98.70% (95%CI: 92.98%-99.97%)
Mutation not detected	26	76		

### LOD and specificity of the AS-RPA/SYBR assay

**Fig 3:**
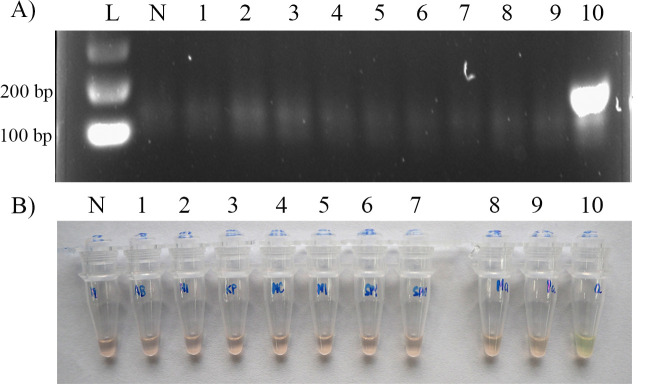
Specificity RPA testing. (A) Agarose gel electrophoresis showed the expected *rpoB*526 RPA product when testing with wild-type *M*. *tuberculosis* H37Rv DNA only (lane 10). Lane L is a 100-bp DNA ladder, lane N is a no-template control, and lanes 1–9 are DNA extracted from *A*. *baumannii*, *H*. *influenzae*, *K*. *pneumoniae*, *M*. *catarrhalis*, *M*. *intracellulare*, *S*. *pyogenes*, *S*. *pneumoniae*, *M*. *avium*, and *P*. *aeruginosa*, respectively. (B) *RpoB*526 RPA products were observed for the SYBR Green I colour change by the naked eye. Tube N is a no-template control, and tubes 1–9 are DNA extracted from *A*. *baumannii*, *H*. *influenzae*, *K*. *pneumoniae*, *M*. *catarrhalis*, *M*. *intracellulare*, *S*. *pyogenes*, *S*. *pneumoniae*, *M*. *avium*, and *P*. *aeruginosa*, respectively, showing an orange colour, which indicates no amplification. Tube 10 is *M*. *tuberculosis* H37Rv DNA, showing a green colour, which implies a positive amplification result.

The LOD of each primer set in the AS-RPA/SYBR assay was 5 ng, except for the IS1081 primers, for which the LOD was 0.05 ng. The AS-RPA/SYBR assay did not cross-react with *A*. *baumannii*, *H*. *influenzae*, *K*. *pneumoniae*, *M*. *catarrhalis*, *P*. *aeruginosa*, *S*. *pneumonia*, *S*. *pyogenes*, *M*. *avium*, or *M*. *intracellulare* (Fig 3).

## Discussion

One primary TB control strategy is early detection and prompt treatment [19]. However, the emergence of MDR-TB is one of the factors that disrupts successful TB control. Rapid and accurate MDR-TB diagnosis could guide appropriate treatment, decreasing morbidity and mortality rates. Hence, we developed a novel, rapid, and easy-setup RPA assay to detect MDR-TB, which can be easily used for routine diagnosis by low-resource-setting laboratories. Our AS-RPA/SYBR assay targeted four major mutations, *rpoB*516, *rpoB*526, *rpoB*531, and *katG*315, associated with rifampicin- and isoniazid-resistant TB.

Codons 531, 526, and 516 are the most common mutation sites among rifampicin-resistant TB reported globally and confer a high level of rifampicin resistance (32 to 256 μg/mL). Other mutations, such as at codons 522 and 533, have been occasionally reported, but they are associated with low-level resistance [20]. A previous study from Thailand showed that all 143 MDR-TB isolates had mutations at *rpoB*, with 98% of strains mutated in the hot-spot RRDR and the remaining isolates mutated in the area of the N-terminus. Mutations at codons 531, 526, and 516 were found the most, with 58%, 25%, and 9% detection, respectively. Codon 526 exhibited the most variable nucleotide substitutions [3]. Isoniazid resistance is mainly associated with mutations in *katG* codon 315 in Thailand and other countries [2, 5]. Our results showed a concordant result with an earlier study. However, the DNA samples tested in this study were derived from *M*. *tuberculosis* colonies isolated from leftover routine specimens. They were not representative of the population attending the hospitals.

Loop-mediated amplification (LAMP) is an isothermal amplification recommended by the WHO for the detection of TB. The LAMP test takes less than 1 hour, and its results can be read by the naked eye under UV light [21]. Previous studies demonstrated that the application of LAMP coupled with Au nanoprobes could detect specific mutation sites related to rifampicin and isoniazid resistance in *M*. *tuberculosis* [22, 23]. RPA is another isothermal amplification that has excellent sensitivity and specificity compared with that of conventional PCR. To the best of our knowledge, there is no evidence comparing RPA to LAMP to directly detect *M*. *tuberculosis* from specimens. However, RPA offers many advantages over other isothermal amplification techniques, such as simple primer design, a short reaction time, a broad temperature range (25–45°C), and multiplexing availability [24]. With a minimal instrumentation requirement and a wide range of storage temperatures (<-15°C, 2–8°C or 22–28°C) and cold chain-independent transportation, RPA is very suitable for basic laboratory settings or as a POCT [13, 25–29].

Rifampicin- and isoniazid-resistant TB occur mainly due to single point mutations in *rpoB* and *katG*, respectively. Therefore, single-nucleotide polymorphism (SNP)-based detection has been implemented in many novel generations of TB diagnostic tools. One simple system is based on allele-specific amplification, which dramatically shortens SNP detection times compared to those of sequencing-based platforms. Allele-specific primers have been used in the RPA reaction to differentiate between wild-type and mutant [15, 27, 30]. Our established RPA applied allele-specific primers combined with SYBR Green I to conduct instrument-free nucleic acid amplification and MDR-TB detection. Other types of mutations such as deletion and insertion can also be detected by allele-specific amplification through intentional multiple base-pair mismatches between primer-template [31]. However, among the different types of mutations, substitution rather than indels is the most significant mutation associated with rifampicin- and isoniazid-resistant TB. Thus, allele-specific primer with additional single-base mismatch exhibits sufficient discriminatory power to differentiate between wild-type and mutant in our recent study.

AS-RPA/SYBR showed a high degree of specificity for MDR-TB diagnosis compared with that for DNA sequencing. All four mutated gene targets corresponding to rifampicin and isoniazid resistance were identified with 100% specificity. The discriminatory power between the wild-type and mutant was increased by adding a single mismatch of three base pairs from the 3’ terminus of the allele-specific primers [18]. Here, each forward allele-specific primer’s furthest 3’ base was designed to specify the wild-type allele expected to anneal. The second base was always annealed to either the wild-type or mutant allele. Furthermore, the third base was an intentional mismatch that will never anneal to either type of allele. Previous studies showed a consistent result between allele-specific RPA and DNA sequencing methods when detecting a single-base mutation [27, 30]. When compared to the phenotypic drug susceptibility test, AS-RPA/SYBR showed 59.38% *katG*315 detection. A *katG*315 mutation was identified in one isoniazid-susceptible strain. However, this mutation was concordant with a result obtained from DNA sequencing.

The sensitivity of the AS-RPA/SYBR assay reported here is high (100% sensitivity) compared to that of DNA sequencing. Compared to a phenotypic drug susceptibility test, sensitivity decreased to 85.19% (for rifampicin) and 59.38% (for isoniazid). Our AS-RPA/SYBR assay focused on detecting mutations only at codons 516, 526, and 531 within the RRDR of the *rpoB* gene and codon 315 of the *katG* gene. Therefore, this assay cannot identify mutations outside of these four target sites. Over 95% of rifampicin-resistant *M*. *tuberculosis* strains have a mutation in the RRDR, and the most prevalent mutations (over 80%) affect codons 531, 526, and 516. Less than 5% of resistant strains do not show a mutation in the RRDR or other regions of the *rpoB* gene [20]. Phenotypic isoniazid resistance has been related to multiple genes but mostly *katG* (60–80%). A previous study from Thailand revealed mutations in *katG*, *inhA*, the *oxyR*-*ahpC* intergenic region, and *ndh* in 80.6%, 13.8%, 2.5%, and 0.6% of cases, respectively. Almost 98% of the *katG* mutations were serine substitution to threonine at codon 315. In addition, one interesting study revealed a higher percentage of Ser315Thr in MDR strains than in isoniazid monoresistant strains [32]. Our AS-RPA/SYBR assay showed the potential to render a rapid and specific method for detecting rifampicin resistance at *rpoB* gene positions 531, 526, and 516 and isoniazid resistance at *katG* gene position 315, which covers the majority of mutations associated with MDR-TB. Any amino acid substitution at these four target sites would not affect the detection efficiency. Other mutated gene targets should be incorporated in the assay to improve the sensitivity in the coverage of additional mutant strains. Besides, a sample size of the most common codon, especially codon 516 and 526, should be expanded to improve the confidence interval of the results.

AS-RPA/SYBR showed no cross-reaction with other bacterial pathogens. The LOD was 5 ng in a 50 μL RPA reaction. A higher sensitivity of 0.05 ng was reported in an RPA coupled with SYBR-based detection of *M*. *tuberculosis* using an IS1081-specific primer in our previous study [16]. The lower sensitivity observed in this study can probably be attributed to the nature of the targeted gene detected. Allele-specific RPA may require template concentrations greater than gene detection for successfully changed SYBR Green I colour observations [27]. Furthermore, the amplicon length might affect the LOD of SYBR Green I. The optimal DNA length recommended for SYBR Green I detection is generally less than 200 bp [33].

RPA has been employed in many studies to detect the *M*. *tuberculosis* genome [13, 16, 34, 35]. However, to the best of our knowledge, only two publications demonstrate MDR-TB detection by RPA integrated by lab-on-a-disc and a fluorescent detector [14, 15]. SYBR Green I was chosen as the detection platform in our recent study to obtain instrument-free nucleic acid amplification and detection for MDR-TB diagnosis. SYBR Green I is a cyanine dye used as a nucleic acid stain for a detection platform in several studies. It preferentially binds to double-stranded DNA, resulting in a DNA-dye complex that emits green light and can be detected by the naked eye [33]. When SYBR Green I was conjoined with our RPA technique, this platform’s outcomes can be achieved within only 30–40 minutes (from sampling to read out) without any specific instrument requirements, which might be suitable for a POCT. In our previous pilot studies, AS-RPA/SYBR was preliminarily validated with a patient sputum sample. The results showed that AS-RPA/SYBR required a high concentration and quality of DNA template. Thus, a proper but straightforward extraction method for a patient sputum sample should be developed. Moreover, AS-RPA/SYBR should be further validated with nucleic acid from the direct specimens to fulfil the demand for a truly simple and rapid diagnosis of MDR-TB.

## Conclusion

Our study demonstrates that AS-RPA/SYBR clearly identified major specific point mutations at codons 531, 526, and 516 of *rpoB* and codon 315 of *katG*, which are associated with rifampicin and isoniazid resistance of *M*. *tuberculosis*, respectively. AS-RPA/SYBR is rapid and easy to perform. Due to the lack of specific and expensive laboratory instrument requirements, AS-RPA/SYBR can be implemented as a new molecular diagnostic method for detecting MDR-TB. The application of AS-RPA/SYBR could limit the spread of MDR-TB, especially in low-resource settings. A larger number of clinical samples should be evaluated to confirm the test’s diagnostic performance and reliably apply it to routine laboratory applications.

## Supporting information

S1 Appendix How to design allele-specific RPA primer. (DOCX)

S1 Raw images (PDF)
