# Supplementary material for: Rapid detection of multidrug-resistant tuberculosis based on allele-specific recombinase polymerase amplification and colorimetric detection
Source: PLoS One. 2021 Jun 11;16(6):e0253235. doi: 10.1371/journal.pone.0253235 (PMC8195408; doi:10.1371/journal.pone.0253235)
Supplement: S1 Appendix — (DOCX) [file pone.0253235.s001.docx]

**S1 Appendix**

**How to design allele-specific RPA primer**

Due to RPA manufacturer’s recommendation, RPA primers of up to 45 bases long especially 30 to 35 nucleotides result in successful amplification, while excessive long primers (more than 45 nucleotides) could lead the formation of secondary structures. Repeating of one particular nucleotide, specifically guanines at the 5' terminus, or many of small repeats should be avoided. GC content should be between 30% and 70% (1).

Mutation at particular codons involved in drug resistance could lead to several types of amino acids substitution. Hence in this study, allele-specific primers were designed to specified with wild-type (*Mtb* ATCC H37Rv) which amplification only occurred when the template was wild-type. Yaku-Bonczyk principle, allele-specific primers design method, was applied to improve the performance of primers to differentiate between mutant and wild-type strains and also to avoid pseudo positive events. The principle suggested that the last nucleotide at the 3' terminus should specific to the SNP, in this case, we designed to specify with wild-type. Additionally, the third nucleotide from the 3' end of primer was intentionally designed mismatch into primer sequences which the nucleotide will never anneal to either wild-type or mutant strains (2, 3) (Fig S1).


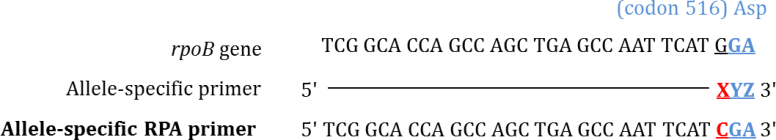


**Fig S1.** **Example of *rpoB*516 allele-specific primer designed by Yaku-Boncynk’s principle.**

Position X was the intentional mismatch nucleotide added into primer sequences, while nucleotide at position Y was designed to always anneal to wild-type or mutant DNA strains. The base at position Z was designed to specify with only wild-type DNA.

Primer screening was recommended by manufacturer to select the best primer sets. To perform the primer screening, two or three forward and reverse primers specific with each codons were designed as candidates. Each candidate was paired and amplified by the TwistAmp^®^ Basic kits for preliminary primer tests. Once the best pairs of primers were identified, and afterward they were used to develop our RPA assays.

Somehow, the results from preliminary screening of allele-specific primers candidates based on Yaku- Bonczyk methods were not satisfying. Redesigning of allele-specific primers by changing the position of mismatch (between last 3 bases at 3' end of primer) could promote the specific amplification.

**Reference**

1. TwistDx. Appendix to the TwistAmp^TM^ reaction kit manuals. Available from: <https://www.twistdx.co.uk/docs/default-source/twistamp-manuals/newappendix_2_.pdf?sfvrsn=8>.

2. Cracolici V. Advanced approach: Introduction to the Yaku-Bonczyk primer design method. Michigan State University; 2011. 38-39 p.

3. Yaku H, Yukimasa T, Nakano S, Sugimoto N, Oka H. Design of allele-specific primers and detection of the human ABO genotyping to avoid the pseudopositive problem. Electrophoresis. 2008;29(20):4130-4140.
