# Supplementary figures and images for: Rapid detection of multidrug-resistant tuberculosis based on allele-specific recombinase polymerase amplification and colorimetric detection
Source: PLoS One. 2021 Jun 11;16(6):e0253235. doi: 10.1371/journal.pone.0253235 (PMC8195408; doi:10.1371/journal.pone.0253235)

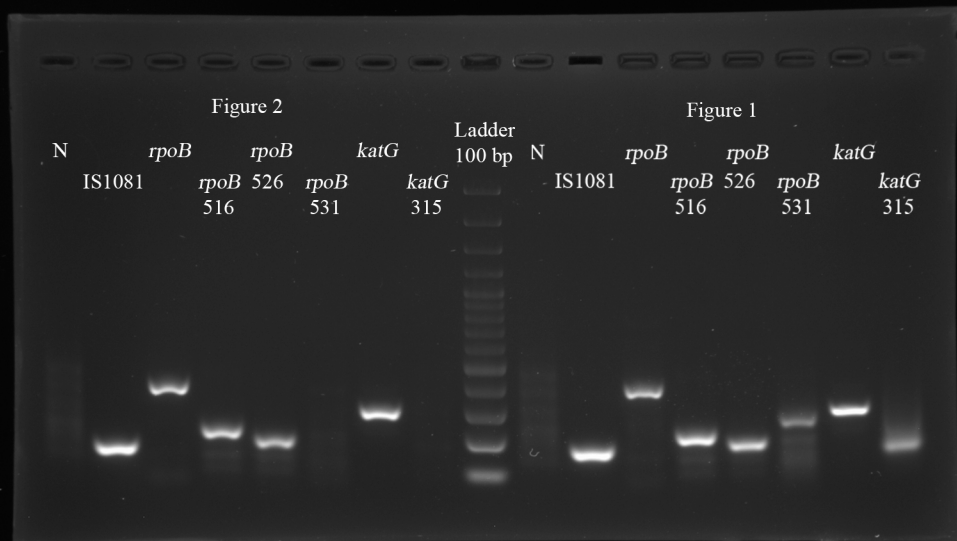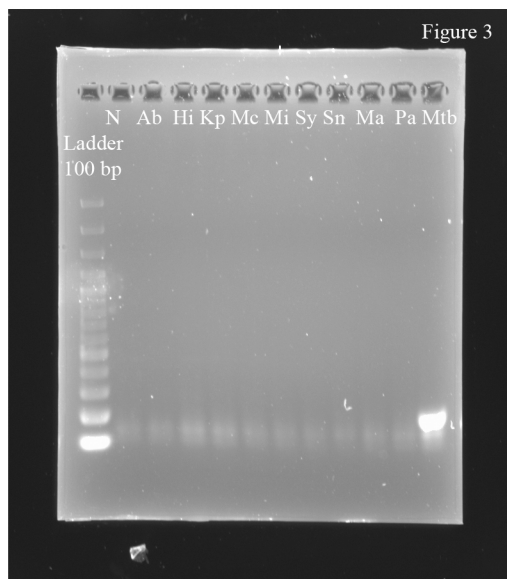

Supplement: S1 Raw images — (PDF) [file pone.0253235.s002.pdf]
